# The IP6K Inhibitor LI-2242 Ameliorates Diet-Induced Obesity, Hyperglycemia, and Hepatic Steatosis in Mice by Improving Cell Metabolism and Insulin Signaling

**DOI:** 10.3390/biom13050868

**Published:** 2023-05-20

**Authors:** Sandip Mukherjee, Molee Chakraborty, Jake Haubner, Glen Ernst, Michael DePasquale, Danielle Carpenter, James C. Barrow, Anutosh Chakraborty

**Affiliations:** 1 Department of Pharmacology and Physiology, Saint Louis University School of Medicine, Saint Louis, MO 63104, USA; 2 Lieber Institute for Brain Development and Department of Pharmacology, Johns Hopkins University School of Medicine, 855 North Wolfe Street Suite 300, Baltimore, MD 21205, USA

**Keywords:** IP6K1, LI-2242, obesity, insulin resistance, NAFLD

## Abstract

Obesity and nonalcoholic fatty liver disease (NAFLD) are global health concerns, and thus, drugs for the long-term treatment of these diseases are urgently needed. We previously discovered that the inositol pyrophosphate biosynthetic enzyme IP6K1 is a target in diet-induced obesity (DIO), insulin resistance, and NAFLD. Moreover, high-throughput screening (HTS) assays and structure−activity relationship (SAR) studies identified LI-2242 as a potent IP6K inhibitor compound. Here, we tested the efficacy of LI-2242 in DIO *WT* C57/BL6J mice. LI-2242 (20 mg/kg/BW daily, i.p.) reduced body weight in DIO mice by specifically reducing the accumulation of body fat. It also improved glycemic parameters and reduced hyperinsulinemia. LI-2242-treated mice displayed reduced the weight of various adipose tissue depots and an increased expression of metabolism- and mitochondrial-energy-oxidation-inducing genes in these tissues. LI-2242 also ameliorated hepatic steatosis by reducing the expression of genes that enhance lipid uptake, lipid stabilization, and lipogenesis. Furthermore, LI-2242 enhances the mitochondrial oxygen consumption rate (OCR) and insulin signaling in adipocytes and hepatocytes in vitro. In conclusion, the pharmacologic inhibition of the inositol pyrophosphate pathway by LI-2242 has therapeutic potential in obesity and NAFLD.

## 1. Introduction

Obesity and insulin resistance enhance the risk of development and progression of nonalcoholic fatty liver disease/steatohepatitis (NAFLD/NASH) [1,2,3]. The prevalence of obesity has increased globally, including the US. In 2016, the direct and indirect medical care costs of obesity in the US were more than USD 480 billion and USD 1.24 trillion, respectively [4]. The number of NAFLD patients is also predicted to increase from 83.1 million (2015) to 100.9 million (2030) [3]. A 5–10% loss in body weight or body fat improves hepatic steatosis and fibrosis in NASH patients with obesity [5]. Thus, drugs that can effectively prevent and treat obesity and NASH are urgently needed [1,6,7,8,9,10]. Unfortunately, success in ameliorating obesity is limited because of the complexity of the disease [11], and no FDA-approved drugs are available to treat NAFLD/NASH [1,2,12]. Yet, encouraging data from clinical trials on the effects of the new anti-obesity drug semaglutide on certain parameters of NAFLD/NASH could enhance the possibility that targeting other relevant pathways may also be useful in obesity and obesity-induced NAFLD/NASH [13].

Inositol hexakisphosphate kinase-1 (IP6K1), an inositol pyrophosphate biosynthetic enzyme, is a target in diet-induced obesity (DIO) and obesity-related metabolic diseases [14,15]. The whole-body and adipocyte-specific deletion of *Ip6k1* ameliorates DIO, insulin resistance, and hepatic steatosis [16,17,18,19,20,21,22]. Moreover, the pan-IP6K inhibitor TNP [N2-(m-trifluoro benzyl), N6-(p-nitrobenzyl)purine] [23,24] ameliorates these diseases [25]. IP6K1 has been implicated in human liver disease, as hepatic IP6K1 is upregulated in NASH, alcoholic cirrhosis [21], and hepatocellular carcinoma patients [26]. Furthermore, whole-body and hepatocyte-specific *Ip6k1* deletion protects mice from Western-diet-induced NAFLD/NASH [21]. Thus, the targeting of IP6K1 should have pleiotropic benefits for human health [14]. 

The IP6K1 inhibitor TNP (Figure S1) is not an ideal lead compound [14,15,25,27]. As TNP is not the product of a rigorous drug development program, it has the following limitations: TNP is a potent inhibitor of human cytochrome P450s and thus has a high probability of causing drug–drug interactions [25]. It has off-target effects such as enhancing Ca^2+^ and Zn^2+^ levels and differentially alters the activity of the extracellular signal-regulated kinase (ERK) [23,28,29,30]. It contains a nitro-aromatic group which is a toxicology structural alert for mutagenicity and hepatotoxicity. Moreover, it possesses a modest IC_50_ and poor aqueous solubility. Thus, there is ongoing research aiming to develop new IP6K inhibitor compounds by modifying TNP or by identifying new chemical classes of IP6K inhibitors [31,32,33,34,35]. In one such study, a high-throughput screening (HTS) assay of 158,410 compounds followed by structure–activity relationship (SAR) optimization identified LI-2242 as a potent IP6K inhibitor compound [33]. Here, we tested the effects of LI-2242 on the metabolic parameters of cells and DIO mice. 

## 2. Materials and Methods

Details of the materials and assay kits are provided in the Supplementary Materials.

Animal studies: All protocols were approved by the Saint Louis University Institutional Animal Care and Use Committee. Mice were housed at 23 °C under 12 h light/dark cycles and fed ad libitum with free access to water. The high-fat diet (HFD) used in this study provides 61.6%, 20.3%, and 18.1% kcals from fats, carbohydrates, and proteins, respectively). Six-week-old *WT* C57BL6J male mice were fed on the HFD for 10 weeks prior to the beginning of drug treatment. Into these mice, the vehicle (DMSO:Tween 80:water—0.5:1:8.5) and LI-2242 were injected daily (i.p. in the morning) for 7 weeks. HFD feeding was continued throughout the experiment. The glucose tolerance test (GTT) was performed in the 1st and 3rd weeks of treatment, and glucagon tolerance (GgTT) was measured in the 4th week of treatment to determine progressive changes in the systemic and liver glycemic profiles of the animals. Food intake was assessed in the 5th week of treatment. The body composition was evaluated before starting the treatment and after 6 weeks of treatment. Energy expenditure was measured in the 7th week of treatment, after which the mice were euthanized. The mice were housed in groups throughout the study except during food intake and the CLAMS studies. Tissues and plasma isolated from the euthanized mice were used for downstream analysis. 

Pharmacokinetic (PK) studies and standardization of the vehicle: PK studies were performed on chow-fed mice at Pharmaron Inc., Waltham, MA, USA. T_1/2_, T_max_, C_max_, AUC_last_, AUC_inf_, AUC__extrap_obs_, MRT_inf_obs_, and AUC_last/D_ parameters were measured in the serum after a single i.p. injection of 20 mg/BW LI-2242 in DMSO:Tween 80:water (0.5:1:8.5). In a separate study, the accumulation of this compound in the liver was measured after three i.p. injections (once daily) of 10 mg/BW LI-2242 in a different vehicle (20% PEG 400 in PBS 0.2 M, pH 7.4). The first vehicle was selected for the in vivo study based on our previous long-term experiments with an IP6K inhibitor [25]. At the end of the drug treatment, concentrations of LI-2242 in the plasma of the DIO mice were also measured.

Body weight and body composition analyses: Body weight was measured weekly by placing the mice on a weighing machine. For body composition analysis, the fat, lean, and fluid mass paramters of the mice were measured using a Minispec LF-NMR (Brucker Optics, Billerica, MA, USA) analyzer [16,19,21]. The instrument was calibrated before the analysis. 

Measurement of blood glucose and the glucose and glucagon tolerance tests (GTT and GgTT): For GTT, glucose (2 g/kg BW, i.p.) was injected into 16 h fasted animals. For GgTT, glucagon (15 µg/kg BW, i.p.) was injected into 5 h fasted mice. The blood glucose levels were measured using a glucometer by pricking the tail veins of the mice before and after the indicated time periods of injection [21]. An average of two readings were recorded. 

Food intake: For this study, mice were singly housed and acclimatized for 2 days. On the day of the experiment, the diet was weighed and then placed in the cage at 6 p.m. The remaining amount of food was weighed the following day at the same time to quantify the 24 h food intake. This process continued for 4 days. The average amount of food consumed per mouse per day was calculated. The mice had full access to drinking water throughout the study [22].

Whole-body energy expenditure, respiratory exchange ratio, and activity profiles: Energy expenditure was measured following a previously published method [16]. Mice were placed individually in metabolic cages at 23 °C with precise thermostatic control in a Comprehensive Laboratory Monitoring System (CLAMS; Columbus Instruments, Columbus, OH, USA) and were acclimatized for 2 days. Afterwards, oxygen consumption, (VO_2_), carbon dioxide release (VCO_2_), and spontaneous locomotor activity were measured for 7 days. Energy expenditure (EE) and the respiratory exchange ratio (RER) were calculated using the following equations: RER = VCO_2_/VO_2_; EE (kcal/h) = (3.815 + 1.232 × RER) × VO_2_. The values were normalized according to lean mass.

Tissue and blood collection and the assessment of serum metabolic and liver injury parameters: Plasma, adipose tissue, liver, and other tissues were isolated from 4 h-fasted mice following standard procedures [16,21,25,36]. The plasma TAG, NEFA, and total cholesterol of euthanized mice were measured at the Mouse Metabolic Phenotyping Centers (MMPC), University of Cincinnati, College of Medicine Pathology & Laboratory Medicine. The serum insulin concentration was determined using an ultra-sensitive mouse ELISA kit [21]. The serum AST and ALT levels were measured using commercial kits [21]. For the Western blot analysis, tissues were flash-frozen in liquid nitrogen and stored at −80 °C until further processing. For the qRT-PCR studies, tissues were stored in RNAlater at 4 °C for 24 h followed by freezing at −80 °C. 

Fecal lipid extraction and TAG assay: Fecal lipids were extracted following a standard protocol [37]. Feces were collected from mice that were singly housed during the food intake study, flash-frozen in liquid nitrogen, and stored at −80 °C until processing. The snap-frozen feces (100 mg per mouse) were dried, pulverized, homogenized, and vortexed with PBS (200 μL) and then mixed with chloroform-methanol (*v*/*v*, 1.2 mL). The mixture was centrifuged, and the organic phase was transferred into a new tube and evaporated until dry. The TAG levels were measured using a commercial kit [21]. 

Hematoxylin and eosin (H&E) staining of tissues: Fat pads and livers from the euthanized mice were fixed in 4% paraformaldehyde and 10% neutral buffered formalin, respectively. The fat pads and livers were stored at 4 °C and RT, respectively, until processing. Fat pad and liver samples were collected from the same areas/lobes of the mice to ensure consistent results. The fixed tissues were paraffin-embedded. The paraffin blocks were trimmed, and serial sections were cut. Deparaffinized sections were gradually dehydrated in ethanol and stained with hematoxylin and eosin (H&E) staining [16,21]. 

Steatosis scoring: H&E-stained liver slides were scored for hepatic steatosis and hepatocyte hypertrophy following a standard semiquantitative scoring method [21,38]. The scoring was denoted as follows: 0 (no steatosis or steatosis covering < 5% of the hepatic parenchyma), 1 (steatosis covering 5–33% of the parenchyma), 2 (33–66% of parenchyma), and 3 (>66% of parenchyma). Hepatocyte enlargement was scored as follows: 0 (none), 1 (mild, 1–2/200 X field), 2 (moderate, 3–4/200 X field), and 3 (severe, >4/200 X field). The percentage of hypertrophy was calculated as described for steatosis. 

RNA isolation and quantitative RT-PCR (qRT-PCR): RNA isolation and qRT-PCR were conducted following a standard ΔΔCt method. Total RNA was extracted from mouse tissues using the RNeasy Lipid Tissue Mini Kit. Reverse transcription was performed using the High-Capacity cDNA Reverse Transcription Kit. Gene expression was determined using the SYBR green method [21]. The expressions of hypoxanthine guanine phosphoribosyl transferase (*Hprt1*) and ribosomal protein lateral stalk subunit P0 (*Rplp0*) were used as controls for the WAT and liver, respectively. The comparative threshold cycle method was used to calculate changes in mRNA abundance.

OCR and ECAR assays of 3T3L1 adipocytes and HepG2 hepatocytes: Cellular OCR and ECAR were measured following a standard protocol [16]. For the adipocytes, 3T3L1 (7000/well) preadipocytes were plated on a manufacturer-provided 96-well plate and differentiated into adipocytes for 7 days [19]. Briefly, 48 h post-confluent preadipocytes were differentiated for 2 days in a differentiation media containing insulin (1 μg/mL), dexamethasone (0.25 μM), 3-isobutyl-1-methylxanthine (0.5 mM), and rosiglitazone (2 μM). After that, the differentiation media was replaced with the maintenance media containing insulin at 1 μg/mL. The media was replaced with fresh media every other day until the cells were fully differentiated on day 8. The post-differentiated adipocytes were serum- and insulin-starved overnight or maintained in serum as indicated. After that, the adipocytes were treated with vehicle or LI-2242 for 3 h (including 1 h in XF media). For the HepG2 cells, 10,000 cells/well were plated as mentioned above. Overnight-serum-starved cells were treated with the indicated concentrations of vehicle or LI-2242, as described for the adipocytes. The OCR and ECAR were measured using a Seahorse Extracellular Flux Analyzer (Seahorse Bioscience, North Billerica, MA, USA) in XF assay medium supplemented with 1 mM pyruvate, 10 mM glucose, and 2 mM glutamine. After the baseline measurements, 3 sequential injections were applied to the wells (indicated as A, B, and C in Figure 5A,E): oligomycin (complex V inhibitor, 1 μM), FCCP (uncoupler, 1 μM), and antimycin A (complex III inhibitor, 0.5 μM) combined with rotenone (complex I inhibitor, 0.5 μM). The basal, coupled, uncoupled, and spare OCRs were calculated following published methods [16].

Insulin signaling studies on 3T3L1 adipocytes and HepG2 hepatocytes: Here, 3T3L1 preadipocytes were differentiated into mature adipocytes as described above. The vehicle and indicated concentrations of LI-2242 were added to overnight-serum-starved adipocytes for 3 h. After that, insulin (10 nM) was added to the media for the indicated time. HepG2 cells were grown according to standard protocols and treated with the vehicle, LI-2242, and insulin, as described above. Following the treatments, the cells were collected and lysed and the protein samples were used for immunoblotting studies. 

Immunoblotting studies: Tissue and cells were lysed in a lysis buffer containing protease–phosphatase inhibitors. The total protein was quantified using a BCA protein assay kit. Equal amounts of total protein were loaded onto 10% SDS-PAGE. The proteins were detected via immunoblotting following standard protocols [19,21]. Equal protein loading was confirmed via immunoblotting for GAPDH. Densitometric analyses of the protein bands were performed using ImageJ software. For the phosphorylation analysis, the band intensity of the phosphorylated protein was normalized against the total level of the same protein. 

Statistics: Trial/prior experiments were used to determine the sample size with statistical power. Animals were excluded in cases where they showed any signs of random sickness. The numbers of mice (*n*) used in the experiments are indicated in the legends. Immunoblots were quantified using ImageJ software (NIH). Data are presented as the mean ± s.e.m within dot plots. Each symbol represents an individual sample. For multiple comparisons, we used one-way or two-way ANOVA with the Holm–Šidák multiple comparison test, and for 2 independent data sets, two-tailed unpaired Student’s *t*-tests were used. Area under curve analysis was conducted and statistical significance was calculated using GraphPad Prism 8.2.1. 

## 3. Results

### 3.1. LI-2242 Ameliorates Obesity in DIO Mice via a Specific Reduction in Body Fat

**Figure 1:**
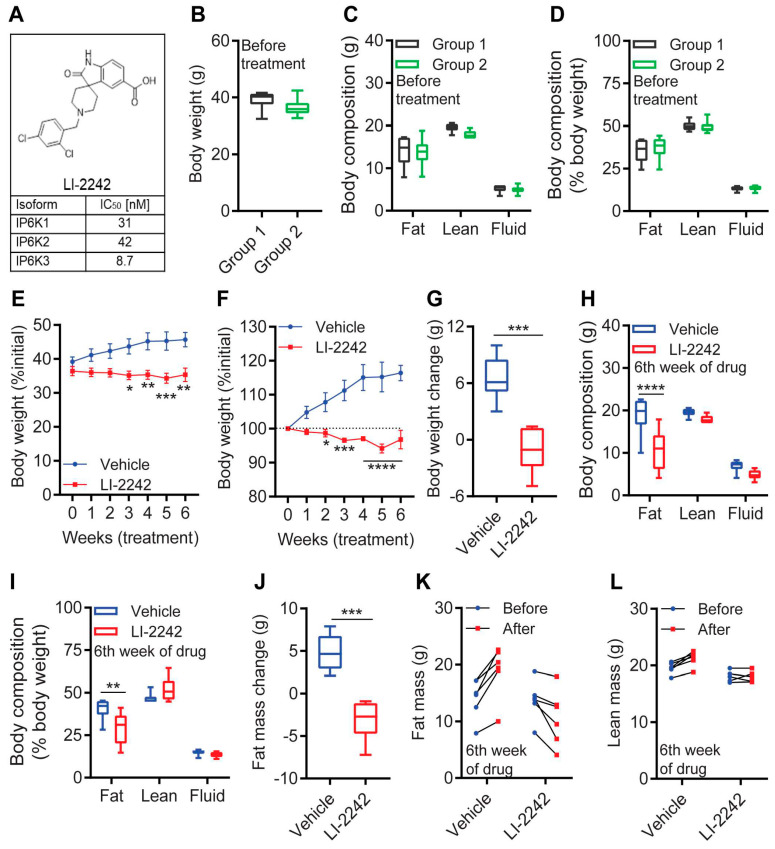
LI-2242 ameliorates obesity in DIO mice via a specific reduction in body fat. (**A**) Structure of LI-2242 and its IC_50_ values for IP6Ks. (**B**) Body weight of DIO mice in groups 1 and 2 prior to the treatment. (**C**,**D**) Total and percent (over total body weight) body compositions of DIO mice in groups 1 and 2 prior to the treatment. (**E**) Weekly body weight of the DIO vehicle- and LI-2242-treated mice. (**F**) Percent change in weekly body weight in the DIO vehicle- and LI-2242-treated mice. (**G**) Change in body weight in the DIO vehicle- and LI-2242-treated mice. (**H**,**I**) Total and percent body compositions of the DIO vehicle- and LI-2242-treated mice. (**J**) Average change in body fat in the DIO vehicle- and LI-2242-treated mice. (**K**) Change in fat mass in each DIO mouse after vehicle and LI-2242 treatment. (**L**) Lean mass of each DIO mouse after vehicle and LI-2242 treatment. *N* = 6 mice were used per cohort. Mean ± s.e.m. shown within plots. For multiple comparisons, two-way ANOVA with the Holm–Šidák multiple comparison test was used, and for two independent data sets, the two-tailed unpaired Student’s *t*-test was used. * *p* < 0.05, ** *p* < 0.01, *** *p* < 0.001, **** *p* < 0.0001.

LI-2242 is a pan-IP6K inhibitor which inhibits all the three isoforms of IP6K at IC_50_ values of 8.7–31 nM [33]. The potency of LI-2242 is much lower than the reported values for TNP (0.25–1 μM) [34] (Figure 1A and Figure S1). Prior to the in vivo efficacy studies, single-dose pharmacokinetics was performed on young, chow-fed, male *WT* C57BL6 mice using 20% PEG 400 in PBS (0.2 M, pH 7.4) as a vehicle. The intraperitoneal (i.p.) dosing of LI-2242 showed good bioavailability (75%) and an extended half-life (11 h) compared to the i.v. dosing (6 h). While the C_max_ after a single 10 mg/kg i.p. dose was high (160 μM), the very high plasma protein binding (99.4%) meant that only 980 nM of free drug was available, and by 24 h, only 21 nM of free drug was left, which was below the in vitro enzyme IC_50_ value of 31 nM. A pilot dose-ranging i.p. PK study on the same young, chow-fed, male *WT* C57BL6 mice at 1, 10, and 100 mg/kg using the same 20% PEG 400 in PBS (0.2 M, pH 7.4) vehicle showed high exposure that was dose-responsive, and accumulation was evident at the 100 mg/kg dose. The tissue distributions at 10 and 100 mg/kg showed a slight 1.4-fold increase in the liver tissue concentration compared to the plasma at both doses (Table S1). The muscle concentrations were much lower than those in the plasma at 10 and 100 mg/kg (0.12- and 0.25-fold lower, respectively). Subsequent studies showed compound precipitation after high doses in the peritoneal cavity following i.p. dosing with the 20% PEG 400 in PBS (0.2 M, pH 7.4) vehicle; therefore, for the pivotal efficacy studies, the dose and vehicle were changed to 20 mg/kg as a 0.5 mg/mL solution of DMSO:Tween-80:water (0.5:1:8.5), which we found to be tolerated in long-term efficacy studies [25]. In lean mice, exposure at this dose and vehicle provides C_max_ and C_24_ values of 173 μM and 7.7 μM, respectively (Table S2). Correcting for extensive plasma protein binding (99.4%) lead to 0.05–1 μM free drug concentrations over a 24 h period, in excess of the in vitro IC_50_ value of 0.031 μM. The concentrations of LI-2242 in the plasma of the DIO mice six hours after the final dose were 46 ± 5 μg/mL, being similar to the pilot PK concentrations in the lean mice at that timepoint (33 ± 24 μg/mL), suggesting similar PK in lean and obese mice, as well as minimal accumulation on repeat dosing.

Next, studies were designed to test the in vivo efficacy of LI-2242 in DIO mice (Figure S2). DIO was developed in *WT* C57BL6J male mice by feeding them on a high-fat diet (HFD) for 10 weeks [16,19,25]. After that, the body weight and composition of the mice were measured, and the mice were divided into two groups: Groups 1 and 2. Both DIO groups showed similar body weights and body compositions at this point (Figure 1B–D). Groups 1 was treated with the vehicle and Group 2 was treated with LI-2242, while HFD-feeding was continued in both groups. During the treatment period, the vehicle-treated mice gained ~6.5 g (~16.5%), but the LI-2242-treated mice lost ~1.1 g (~3.02%) of body weight (Figure 1E,F). Thus, after six weeks of treatment, the LI-2242-treated mice showed a decrease of ~7.6 g in body weight (Figure 1G). Body composition analysis revealed that the reduction in body weight in the LI-2242-treated mice was primarily due to a significant reduction in fat mass (Figure 1H). Accordingly, the average percent fat mass (over the total body weight) was significantly decreased, whereas the percent lean mass and percent fluid mass were largely unaltered in the LI-2242-treated mice compared to the vehicle-treated mice (Figure 1I). Overall, the vehicle-treated mice gained ~4.8 g fat mass, but LI-2242-treated mice lost ~3.1 g fat mass, resulting in a drug-induced reduction of ~7.9 g in fat mass (Figure 1J). Each mouse from the vehicle or LI-2242 group showed the same trend in the alteration of body fat (Figure 1K). The lean and fluid masses were slightly increased in the vehicle-treated mice, but they remained the same in the LI-2242-treated mice (Figure 1L and Figure S3). Thus, LI-2242 treatment reduces body weight in DIO mice primarily by reducing fat mass, without substantially altering lean and fluid mass. 

### 3.2. LI-2242 Improves Glycemic Parameters in DIO Mice

**Figure 2:**
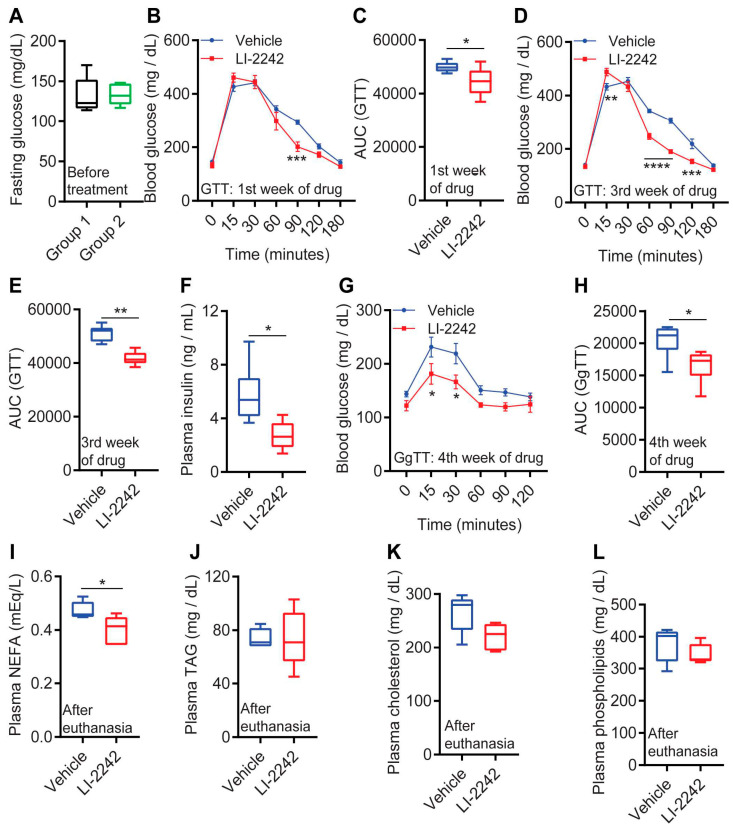
LI-2242 improves glycemic parameters in DIO mice. (**A**) Levels of blood glucose in overnight-fasted DIO mice in groups 1 and 2 prior to the treatment. (**B**,**C**) Glucose tolerance test (GTT) of the DIO vehicle- and LI-2242-treated mice after 1 week of treatment. (**D**,**E**) GTT in DIO vehicle- and LI-2242-treated mice after 3 weeks of treatment. (**F**) Levels of plasma insulin in DIO vehicle- and LI-2242-treated mice after the conclusion of the study. (**G**,**H**) Glucagon tolerance test (GgTT) of DIO vehicle- and LI-2242-treated mice after 4 weeks of treatment. (**I**–**L**) Plasma lipid profiles of DIO vehicle- and LI-2242-treated mice after the conclusion of the study (7 weeks of treatment). *N* = 6 mice per cohort were used in (**A**–**H**), and *N* = 5 mice were used in (**I**–**L**). Mean ± s.e.m. shown within plots. For multiple comparisons, two-way ANOVA with the Holm–Šidák multiple comparison test was used, and for two independent data sets, the two-tailed unpaired Student’s *t*-test was used. * *p* < 0.05, ** *p* < 0.01, *** *p* < 0.001, **** *p* < 0.0001.

Next, we tested the effects of LI-2242 treatment on the glycemic parameters of the DIO mice. The blood glucose levels of overnight-fasted-animals prior to the treatment indicated that HFD feeding caused similar levels of hyperglycemia in both groups (Figure 2A). After 1 week of treatment, clearance of the exogenous glucose from the blood (glucose tolerance test—GTT) was slightly faster in the LI-2242-treated mice compared to the vehicle-treated mice (Figure 2B,C). Moreover, in the 3rd week of treatment, the LI-2242-treated mice exhibited a substantially improved rate of glucose clearance (Figure 2D,E). These results, together with the reduced levels of plasma insulin in the LI-2242-treated mice (Figure 2F), indicated improved insulin sensitivity in the drug-treated animals. Disruption of the IP6K1 pathway reduces hepatic gluconeogenesis [21]. Accordingly, the glucagon-induced increase in blood glucose levels was lower in the LI-2242-treated mice (Figure 2G,H). LI-2242 treatment significantly reduced the levels of non-esterified fatty acids (NEFA) in the plasma, whereas the levels of other metabolites such as triglycerides (TAG), cholesterol, and phospholipids were similar in both cohorts (Figure 2I–L). Thus, LI-2242 improves glycemic parameters in DIO mice.

### 3.3. LI-2242 Treatment Improves Metabolic Parameters in the Adipose Tissue of DIO Mice

**Figure 3:**
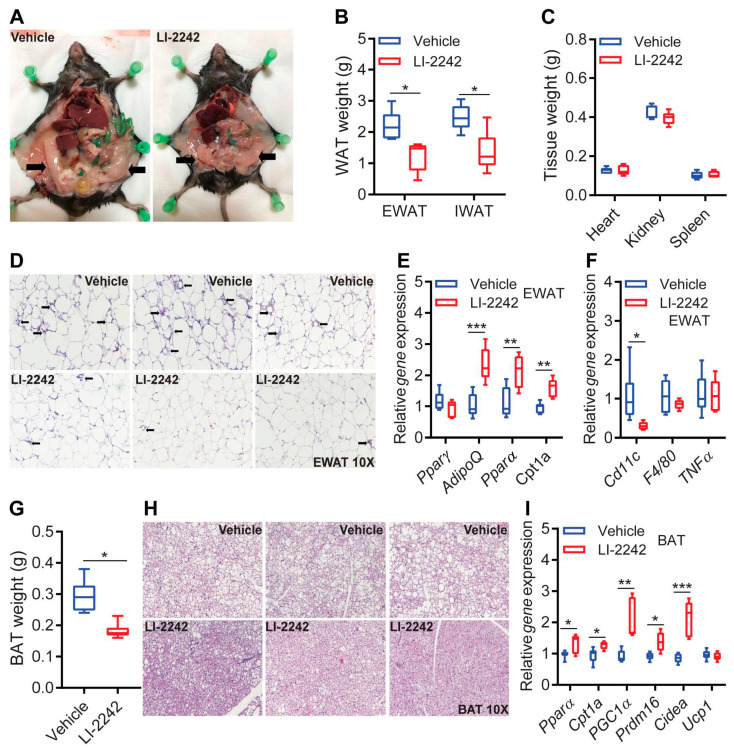
LI-2242 treatment improves metabolic parameters in the adipose tissue of DIO mice. All the parameters were measured in samples isolated from euthanized mice after the conclusion of the study. (**A**) Representative images of DIO vehicle- and LI-2242-treated mice after the conclusion of the study. Arrows indicate fat depots. (**B**) Weight of EWAT and IWAT in DIO vehicle- and LI-2242-treated mice. (**C**) Weight of the heart, kidney, and spleen from DIO vehicle- and LI-2242-treated mice. (**D**) Representative histological images of the EWAT of DIO vehicle- and LI-2242-treated mice. Arrows indicate crown-like structures or inflammatory macrophage accumulation. (**E**) Expression profiles of metabolic genes in the EWAT of DIO vehicle- and LI-2242-treated mice. (**F**) Expression profiles of inflammatory genes in the EWAT of DIO vehicle- and LI-2242-treated mice. (**G**) Weight of BAT in DIO vehicle- and LI-2242-treated mice. (**H**) Representative histological images of the BAT of DIO vehicle- and LI-2242-treated mice. Unstained white spots indicate that lipid droplets accumulated in these areas. (**I**) Expression profiles of metabolic genes in the BAT of DIO vehicle- and LI-2242-treated mice. *N* = 5–6 mice per cohort were used. Mean ± s.e.m. shown within plots. For multiple comparisons, two-way ANOVA with the Holm–Šidák multiple comparison test was used, and for two independent data sets, the two-tailed unpaired Student’s *t*-test was used. * *p* < 0.05, ** *p* < 0.01, *** *p* < 0.001.

IP6K1 regulates systemic metabolism and insulin signaling primarily by modulating metabolic functions of the adipose tissue and liver [16,17,19,21]. Therefore, we compared the metabolic parameters of these tissues in the vehicle- and LI-2242-treated mice. The drug-treated mice appeared to be leaner and healthier, with a reduced accumulation of fat in the body (Figure 3A, arrows indicate fat). Accordingly, the weight of white adipose tissue depots (epididymal and inguinal—EWAT and IWAT, respectively) was reduced in the LI-2242-treated mice (Figure 3B). The weights of other organs such as the heart, kidney, and spleen were similar in both cohorts, further indicating that the reduction in body weight is specific to fat loss (Figure 3C). The average size of the adipocytes in the EWAT of the LI-2242-treated mice was slightly smaller, indicating adipocyte hyperplasia and improved adipose tissue health (Figure 3D). Accordingly, the gene expressions of the insulin-sensitizing adipokine known as adiponectin (*AdipoQ*), the lipid-metabolism-augmenting transcription factor known as peroxisome proliferator-activated receptor alpha (*Pparα*), and the mitochondrial fatty acid transport protein known as carnitine palmitoyltransferase 1a (*Cpt1a*) were upregulated in the EWAT of the LI-2242-treated mice (Figure 3E). The expressions of the adipogenic transcription factor *Pparγ* were similar in both groups (Figure 3E). Moreover, crown-like structures surrounding the adipocytes, indicative of inflammatory and insulin-resistance-augmenting macrophage accumulation in dysfunctional adipose tissue, were less prevalent in the LI-2242-treated mice (Figure 3D). Consequently, the gene expression of the inflammatory macrophage marker known as cluster of differentiation 11c (*Cd11c*) was decreased in the EWAT of the LI-2242-treated mice (Figure 3F). The expressions of the general macrophage marker EGF-like module-containing mucin-like hormone receptor-like 1 (*F4/80*) and the inflammatory cytokine tumor necrosis factor alpha (*Tnfα*) were similar in both cohorts (Figure 3F). 

Mitochondria-enriched brown adipose tissue (BAT) facilitates energy expenditure through both ATP-coupled and -uncoupled mechanisms and improves insulin sensitivity [39]. LI-2242 treatment reduced the weight of the brown adipose tissue (BAT) due to the reduced accumulation of fat in this depot (Figure 3G,H). Hence, elements of the mitochondrial energy expenditure machinery such as *Pparα*, *Cpt1a*, the transcriptional co-activator known as peroxisome proliferator-activated receptor-gamma coactivator alpha (*Pgc1α*), PR domain containing 16 (*Prdm16*), and cell-death-inducing DNA fragmentation factor-like effector A (*Cidea*) were upregulated in the BAT of the LI-2242-treated mice (Figure 3I). However, the gene expressions of the thermogenic uncoupling protein 1 (*Ucp1*) were similar in both cohorts (Figure 3I). Thus, LI-2242 generally improves the metabolic parameters of adipose tissue in DIO mice.

### 3.4. LI-2242 Treatment Ameliorates Steatosis, Insulin Resistance, and Injury of the Liver in DIO Mice

**Figure 4:**
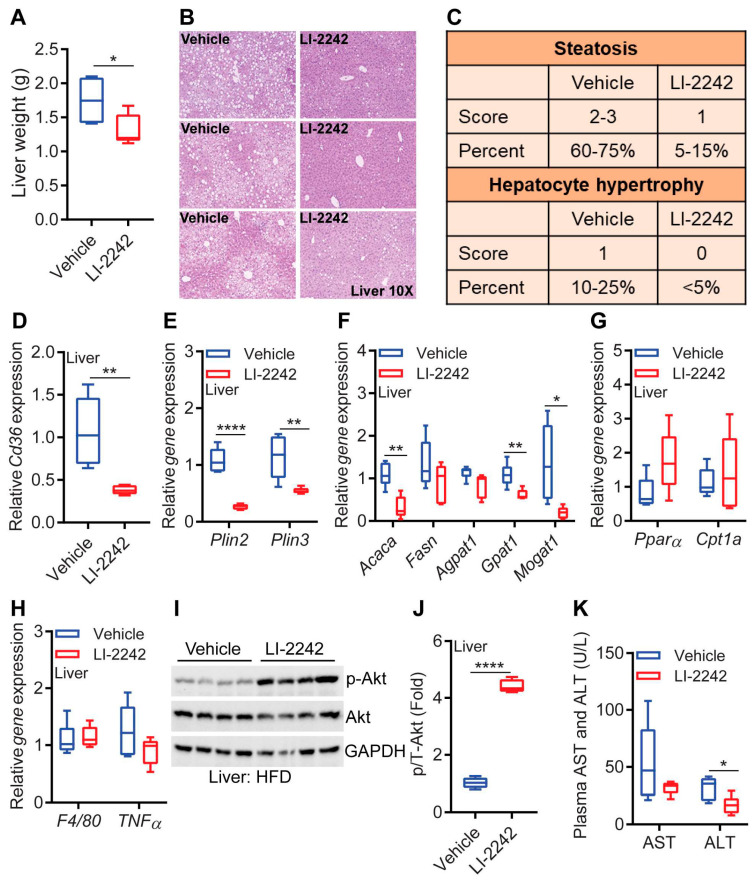
LI-2242 treatment ameliorates steatosis, insulin resistance, and injury of the liver in DIO mice. All the parameters were measured using samples isolated from euthanized mice after the conclusion of the study. (**A**) Weight of livers from DIO vehicle- and LI-2242-treated mice. (**B**) Representative histological images of livers from DIO vehicle- and LI-2242-treated mice. Unstained white spots indicate that lipid droplets accumulated in these areas. (**C**) Scoring for hepatic steatosis and hepatocyte hypertrophy. A score of 1 denotes steatosis covering 5–33% of the parenchyma, whereas scores of 2–3 denote steatosis occupying 33–66% and >66% of parenchyma, respectively. Hypertrophic hepatocytes were scored similarly. (**D**) Gene expression of *Cd36* in the livers of DIO vehicle- and LI-2242-treated mice. (**E**) Gene expression profiles of *Plin2* and *Plin3* in the livers of DIO vehicle- and LI-2242-treated mice. (**F**) Expression profiles of lipogenic genes in the livers of DIO vehicle- and LI-2242-treated mice. (**G**) Gene expression profiles of *Pparα* and *Cpt1a* in the livers of DIO vehicle- and LI-2242-treated mice. (**H**) Expression profiles of inflammatory genes in the livers of DIO vehicle- and LI-2242-treated mice. (**I**) Stimulatory phosphorylation (S473) levels of Akt in the livers of DIO vehicle- and LI-2242-treated mice. Each band represents a different mouse. (**J**) Quantification of p-Akt (over total Akt) from (**I**). (**K**) Levels of the liver injury markers AST and ALT in the plasma of DIO vehicle- and LI-2242-treated mice. *N* = 4–6 mice per cohort were used. Mean ± s.e.m. shown within plots. For multiple comparisons, two-way ANOVA with Holm–Šidák multiple comparison test was used, and for two independent data sets, the two-tailed unpaired Student’s *t*-test was used. * *p* < 0.05, ** *p* < 0.01, **** *p* < 0.0001.

The livers of the LI-2242-treated mice appeared to be smaller and healthier (Figure 3A and Figure 4A). The histological images showed that the reduced liver weight in the LI-2242-treated mice was due to reduced fat accumulation (Figure 4B). The scoring of histological slides for the quantification of hepatic steatosis and hepatocyte hypertrophy showed that both parameters were substantially reduced in the drug-treated mice (Figure 4C). 

The expression of the lipid uptake gene, cluster of differentiation 36 (*Cd36*), was dramatically reduced in the livers of the LI-2242-treated mice (Figure 4D). The hepatic expressions of perlipin 2 and 3 (*Plin2* and *Plin3*), which promote lipid droplet stabilization and lipid storage, were also downregulated in the LI-2242-treated mice (Figure 4E). Among the lipogenic genes, acetyl CoA carboxylase (*Acaca*), the rate-limiting enzyme in fatty acid biosynthesis (generates malonyl CoA from acetyl CoA), and glycerol-3-phosphate dehydrogenase (*Gpat*), the rate-limiting enzyme in de novo glycerolipid synthesis (converts glycerol-3-phosphate and long-chain fatty acyl-CoA to lysophosphatidic acid), as well as monoacylglycerol acyltransferase (*Mogat1*), which generates diacyl glycerol from monoacyl glycerol, were downregulated in the livers of the LI-2242-treated mice (Figure 4F). The gene expressions of other enzymes in the lipogenic pathway, such as fatty acid synthase (*Fasn*) and 1-acylglycerol-3-phosphate-O-acyltransferase 1 (*Agpat1*), were similar in both cohorts (Figure 4F). The gene expressions of *Pparα* and *Cpt1a* were largely similar in the livers of both cohorts (Figure 4G). The hepatic expressions of the macrophage markers *F4/80* and *Tnfα* were also similar in both cohorts (Figure 4H). Increased insulin sensitivity in the LI-2242-treated HFD-fed mice was evidenced by increased stimulatory phosphorylation (S473) of the insulin effector protein kinase Akt in the livers of the drug-treated animals (Figure 4I,J). Finally, we measured the levels of the liver injury marker enzymes aspartate transaminase (AST) and alanine aminotransferase (ALT) in the plasma to assess whether LI-2242 treatment reduced steatosis-induced liver injury in the mice. ALT, but not AST, was significantly reduced in the LI-2242-treated mice (Figure 4K). Thus, LI-2242 treatment ameliorates hepatic steatosis and insulin resistance, protecting DIO mice from liver injury.

### 3.5. LI-2242 Alters the Metabolic Parameters of 3T3L1 Adipocytes and HepG2 Hepatocytes

**Figure 5:**
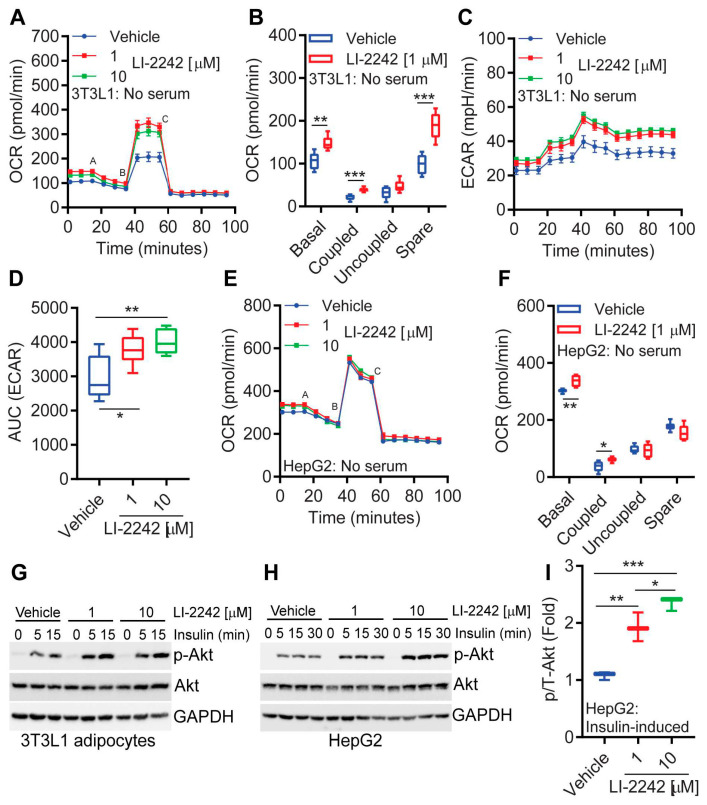
LI-2242 alters the metabolic parameters of adipocytes and hepatocytes in vitro. (**A**,**B**) Basal, ATP-coupled, uncoupled, and spare mitochondrial OCRs of overnight-serum-starved 3T3L1 adipocytes following vehicle and LI-2242 treatments. a–c indicate the injection of oligomycin (complex V inhibitor, 1 μM), FCCP (uncoupler, 1 μM), and antimycin A (complex III inhibitor, 0.5 μM) combined with rotenone (complex I inhibitor, 0.5 μM). Data represent an average of 6 individual wells. (**C**,**D**) ECAR (an indication of glycolysis) in 3T3L1 adipocytes under the above experimental conditions. Data represent an average of 6 individual wells. (**E**,**F**) OCR of overnight-serum-starved HepG2 cells following vehicle and LI-2242 treatments. Data represent an average of 6 individual wells. (**G**) Insulin-induced Akt phosphorylation in vehicle- and LI-2242-treated 3T3L1 adipocytes. Data represent 3 independent experiments. (**H**,**I**) Insulin-induced Akt phosphorylation in vehicle- and LI-2242-treated HepG2 cells. Quantification was performed by comparing the average insulin-induced phosphorylation of Akt at all the time points between the vehicle- and LI-2242 treated samples. Mean ± s.e.m. shown within plots. For multiple comparisons, one-way or two-way ANOVA with the Holm–Šidák multiple comparison test was used, and for two independent data sets, two-tailed unpaired Student’s *t*-tests were used. * *p* < 0.05, ** *p* < 0.01, *** *p* < 0.001.

We assessed the effects of LI-2242 on 3T3L1 adipocytes and HepG2 hepatocytes in vitro. The basal, ATP-coupled, and spare mitochondrial oxygen consumption rates (OCRs) were higher in drug-treated, overnight-serum-starved-adipocytes (Figure 5A,B). The extracellular acidification rate (ECAR), an indication of glycolysis, was also increased in the LI-2242-treated adipocytes in this condition (Figure 5C,D). These parameters were largely similar in the vehicle- and LI-2242-treated cells in serum-containing conditions (Figures S4–S6), indicating that LI-2242 maintains energy expenditure in catabolic conditions. In the HepG2 hepatocytes, LI-2242 treatment enhanced the basal and ATP-coupled OCRs (Figure 5E,F). Moreover, LI-2242 (3 h) treatment enhanced the acute insulin-induced stimulatory phosphorylation (S473) of Akt in the 3T3L1 adipocytes and HepG2 cells (Figure 5G–I). Thus, LI-2242 reduces fat accumulation by augmenting energy expenditure and enhances insulin signaling in a cell-autonomous manner. 

### 3.6. Profiles of the Intake, Absorption, and Expenditure of Energy for LI-2242 Treated Mice

**Figure 6:**
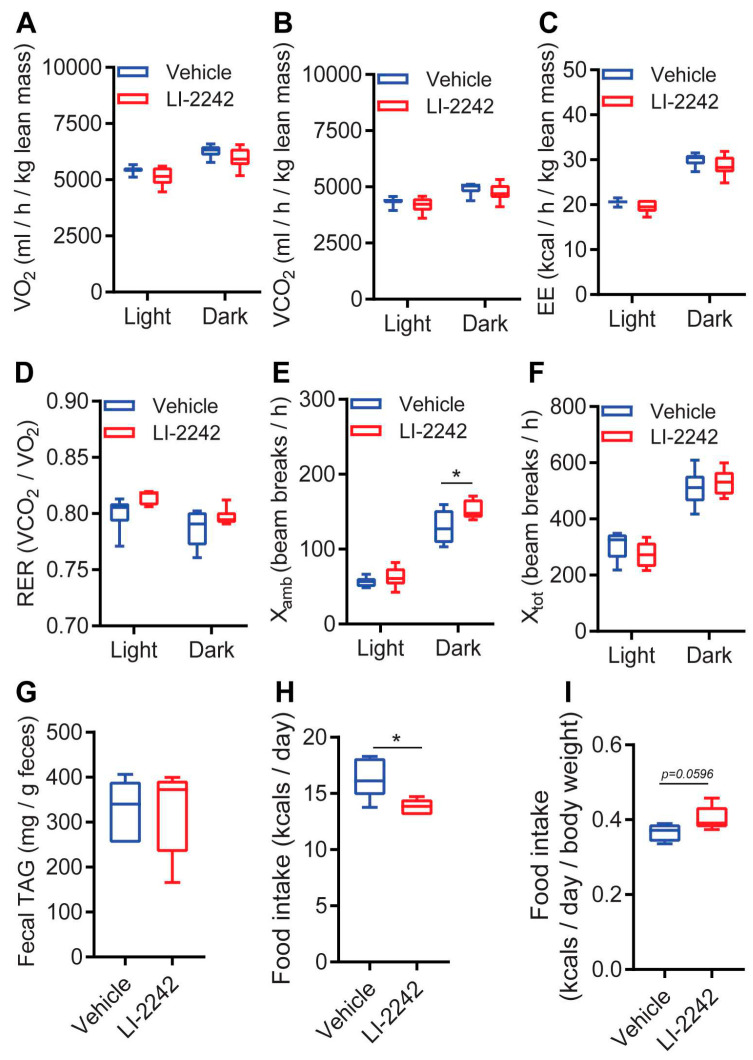
Profiles of the intake, absorption, and expenditure of energy in LI-2242 treated DIO mice. (**A**,**B**) VO_2_ and VCO_2_ in vehicle- and LI-2242-treated mice. (**C**,**D**). EE and RER in vehicle- and LI-2242-treated mice. (**E**,**F**) X_tot_ and X_amb_ activities of vehicle- and LI-2242-treated mice. (**G**) Levels of excretory TAG in vehicle- and LI-2242-treated mice. (**H**,**I**) Total and body-weight-normalized food intakes of vehicle- and LI-2242-treated mice. *N* = 6 mice per cohort were used in (**A**–**F**). *N* = 5–6 mice per cohort were used in (**G**–**I**). Mean ± s.e.m. shown within plots. For two independent data sets, two-tailed unpaired Student’s *t*-tests were used. * *p* < 0.05. For CLAMS, the average values of VO_2_, VCO_2_, and EE were normalized according to lean mass, as lean mass was similar in both cohorts. Statistical significance of the average values for VO_2_, VCO_2_, and EE were calculated using the two-tailed unpaired Student’s *t*-test.

Finally, we compared energy expenditure (EE), absorption, and intake between the DIO vehicle- and LI-2242-treated mice. Oxygen consumption (VO_2_), carbon dioxide release (VCO_2_), and energy expenditure (EE) were similar in both cohorts (Figure 6A–C). The respiratory exchange ratios (RER) were also similar for the vehicle- and LI-2242-treated mice, indicating that the drug did not alter the preference for carbohydrate or fat as a fuel source in the DIO animals (Figure 6D). Night-time ambulatory activity (X_amb_) was slightly higher in the LI-2242-treated mice, although total activity (X_tot_) was similar in both cohorts (Figure 6E,F). LI-2242 treatment did not alter fat absorption, as the excretory levels of triglycerides (fecal TAG) were similar in both cohorts (Figure 6G). The total caloric intake was slightly reduced in the LI-2242-treated mice (Figure 6H). However, when normalized to body weight, the energy intake was found to be marginally higher (Figure 6I).

## 4. Discussion

LI-2242 was chosen for this in vivo study due to its potency, selectivity, and pharmacokinetic profile. Across the three isoforms of IP6K, it demonstrated potent kinase inhibition with IC_50_ values between 9 and 42 nM. It showed <50% inhibition at 10 μM against a panel of 58 representative kinases; however, some inhibition of IPMK (IC_50_ 2 μM) was previously noted [33]. The 20 mg/kg dose was carefully chosen to ensure free drug concentrations over the IC_50_ for most of the 24 h dosing interval in the chow-fed mice, and this was confirmed by measuring the LI-2242 concentrations in the terminal plasma samples at the end of the DIO study. LI-2242 ameliorates obesity, insulin resistance, hepatic steatosis, and liver injury in DIO mice. The treatment specifically reduced fat mass without altering lean mass in the DIO animals. It reduced the expression of genes that enhance fatty acid uptake, TAG synthesis, and lipid droplet stabilization in the liver and upregulated genes that trigger energy expenditure in the adipose tissue. The drug reduced fat accumulation in adipocytes. Moreover, LI-2242 enhanced energy expenditure and insulin signaling in a cell-autonomous manner.

LI-2242 treatment did not cause any noticeable unhealthy behaviors, as the food intake, excretion, and activity parameters were largely normal in these mice. In fact, LI-2242 treatment increased ambulatory activity in the DIO mice. Increased ambulatory activity has been shown to be associated with reduced fat mass [40]. It is not clear whether the inhibition of the IP6K1 isoform in the adipose tissue and liver and/or the skeletal-muscle-enriched IP6K3 isoform increased ambulatory activity or, alternatively, the ambulatory activity was increased due to the overall leanness and improved health of the drug-treated mice. The genetic deletion of *Ip6k1* or pan-inhibition of IP6K isoforms does not have a major impact on energy intake during HFD feeding [17]. In this study, the total caloric intake was slightly reduced in the LI-2242-treated DIO mice. However, the body-weight-normalized food intake was marginally higher in these cohorts. It is conceivable that the food intake was adjusted due to the diminished energy demand of the drug-treated mice, as they were smaller. LI-2242 did not alter the levels of fecal TAG in the DIO mice. IP6Ks are expressed in the brain, and they have pleiotropic effects on brain functions [41,42,43,44,45]; however, LI-2242 does not enter the brain at appreciable levels due to the blood–brain barrier. Moreover, *Ip6k1* deletion reduces serum levels of the food intake inhibitory adipokine, leptin, possibly due to enhanced leptin sensitivity [19]. IP6Ks are also expressed in the gastrointestinal tract [46,47]. Thus, further studies are required to determine the isoform-specific roles of IP6Ks in energy intake and absorption and the impacts of various doses and durations of isoform-specific IP6K inhibitors on these processes.

The deletion of *Ip6k1* or inhibition of the IP6K pathway by the pan-IP6K inhibitor TNP enhances whole-body EE [16,17,19,25], whereas hepatocyte-specific *Ip6k1* deletion does not influence EE in vivo [21]. The EE was similar in the vehicle- and LI-2242-treated mice. In the TNP study, the inhibitor treatment was initiated at the onset of obesity (prevention study), and EE was measured in the 5th week of HFD feeding and drug treatment [25]. Conversely, this was a reversal study, in which mice were fed on the HFD for 10 weeks, followed by LI-2242 treatment along with HFD for 6 weeks, after which EE was measured. LI-2242 induced substantial fat loss. Thus, it is conceivable that after 6 weeks of treatment, EE was adjusted in the LI-2242-treated mice to prevent further weight loss. We measured EE only at this timepoint; thus, conclusions about earlier points cannot be made. Encouragingly, LI-2242 (1 μM) enhanced the coupled OCR and glycolysis in adipocytes in vitro. The EE-enhancing properties of LI-2242 were milder and different than those of *Ip6k1*-deleted or TNP-treated adipocytes and hepatocytes, as these conditions increased the OCR primarily due to increased uncoupling [16,21]. Although the mechanisms of these differences are not clear at this point, a mild increase in EE may preserve mitochondrial functions while reducing fat mass during long-term treatments. Nevertheless, further studies are required to determine the effects of acute and/or short-term treatment with LI-2242 on coupled and uncoupled EE in acute and chronic DIO mice under ambient and thermoneutral temperature conditions and in metabolic cells.

Future studies are required to determine the selectivity of LI-2242 towards IP6K over other kinases, channels, receptors, etc. Based on the outcomes, structure–activity relationship (SAR) studies of LI-2242 should be performed to enhance the selectivity of this compound towards IP6Ks. Activities of the drug-metabolizing enzymes cytochrome P-450s are altered by high-fat diet feeding [48]. TNP inhibits this group of enzymes [25], which is an off-target effect of this non-selective IP6K inhibitor compound, as other classes of potent IP6K inhibitors are largely ineffective [49]. LI-2242 and TNP are structurally distinct; therefore, it is unlikely that LI-2242 inhibits cytochrome P450s. Yet, the pharmacokinetic and pharmacodynamic (PK/PD) properties of LI-2242 and its analogs, including cytochrome P-450 inhibition, should be assessed in chow- and HFD-fed mice before this class of compounds can move to the next stage of drug development.

IP6Ks are recently becoming appreciated as a target for diseases [14,15]. *Ip6k1* deletion has been shown to have beneficial effects on diet- and age-induced obesity, insulin resistance, NAFLD/NASH, osteoporosis, pneumonia, and thromboembolism [16,17,19,20,21,22,50,51]. The deletion of the skeletal-muscle-enriched isoform *Ip6k3* reduces blood glucose, insulin levels, and fat mass and extends the lifespan [46]. Moreover, rodent studies using different classes of IP6K inhibitors have suggested that the inhibition of this pathway has beneficial effects on obesity, insulin resistance, hepatic steatosis, osteoporosis, ischemic/reperfusion injury, chronic kidney diseases, and pneumonia [25,34,49,50,52,53]. Thus, LI-2242 may have beneficial effects on metabolic and other diseases. The dramatic reduction in body weight induced by the current dose (20 mg/kg BW, daily i.p.) suggests that a lower dose may also work for long-term use to avoid possible off-target effects. Future studies will optimize the dose of LI-2242 for long-term studies of rodents. Structure–activity relationship studies (SAR) of LI-2242 will also be required to develop improved IP6K inhibitors and perhaps isoform-selective inhibitors that can be used to treat metabolic diseases.

## 5. Conclusions

The potent pan-IP6K inhibitor LI-2242 ameliorates obesity, insulin resistance, and hepatic steatosis in DIO mice via a specific reduction in body fat and the improvement of insulin sensitivity.

## Data Availability

Data is contained within the article and Supplementary Material.

## Supplementary Materials

The following supporting information can be downloaded at: https://www.mdpi.com/article/10.3390/biom13050868/s1. Figure S1: Reported structure and potency of TNP. Details and references are in the text; Figure S2: Study design to test in vivo efficacy of LI-2242 in DIO mice; Figure S3: Fluid mass of DIO mice before and after vehicle and LI-2242 treatment; Figure S4: Mitochondrial OCR in vehicle- and LI-2242 [1 μM]-treated 3T3L1 adipocytes under serum-containing conditions; Figures S5–S6: ECAR in vehicle- and LI-2242 [1 μM]-treated 3T3L1 adipocytes under serum containing conditions; Tables S1 and S2: Pharmacokinetic of LI-2242.
