# Supplementary material for: The IP6K Inhibitor LI-2242 Ameliorates Diet-Induced Obesity, Hyperglycemia, and Hepatic Steatosis in Mice by Improving Cell Metabolism and Insulin Signaling
Source: Biomolecules. 2023 May 20;13(5):868. doi: 10.3390/biom13050868 (PMC10216446; doi:10.3390/biom13050868)
Supplement: Supplementary file 1 [file biomolecules-13-00868-s001.zip › biomolecules-2354802-supplementary.pdf]

<u>Supplementary figures, tables, and materials</u>

The IP6K inhibitor LI-2242 ameliorates diet-induced obesity, hyperglycemia, and hepatic steatosis in mice by improving cell metabolism and insulin signaling by Mukherjee et al.

Figure S1

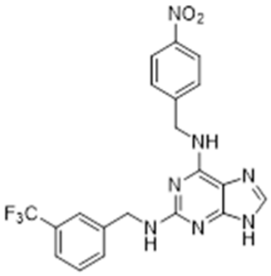

TNP

Isoform	IC_{50} [nM]
IP6K1	270
IP6K2	850
IP6K3	260

Figure S1: Reported structure and potency of TNP. Details and references are in the text.

Figure S2

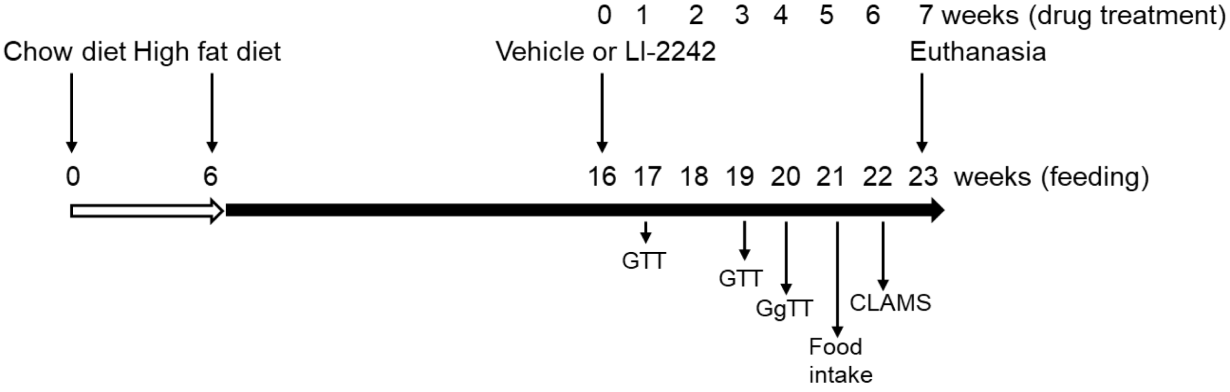

Figure S2: Study design to test in vivo efficacy of LI-2242 in DIO mice.

Figure S3

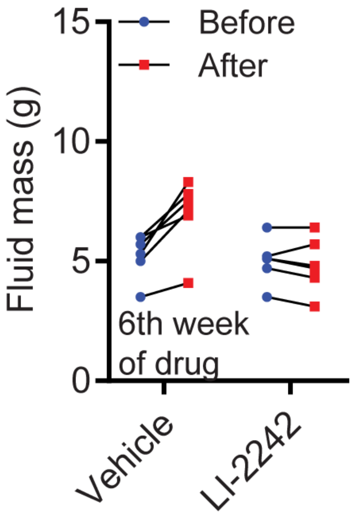

Figure S3: Fluid mass of DIO mice before and after vehicle and LI-2242 treatments.

Figure S4

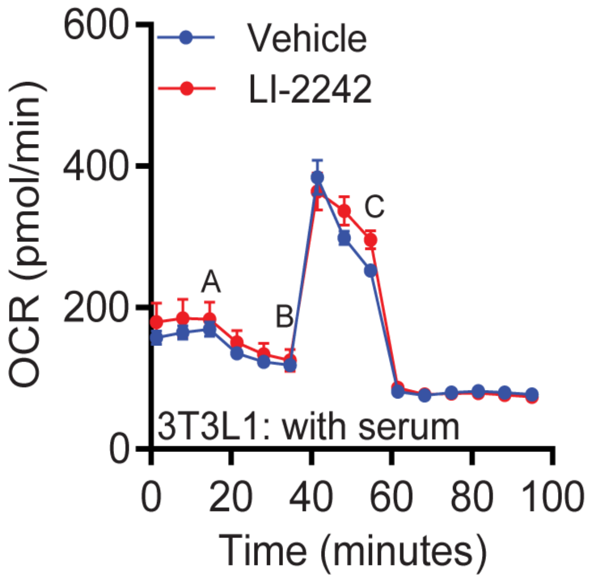

Figure S4: Mitochondrial OCR in vehicle- and LI-2242 [1 μM]-treated 3T3L1 adipocytes under serum-containing conditions. Data represents an average of 7 individual wells.

Figure S5 and S6

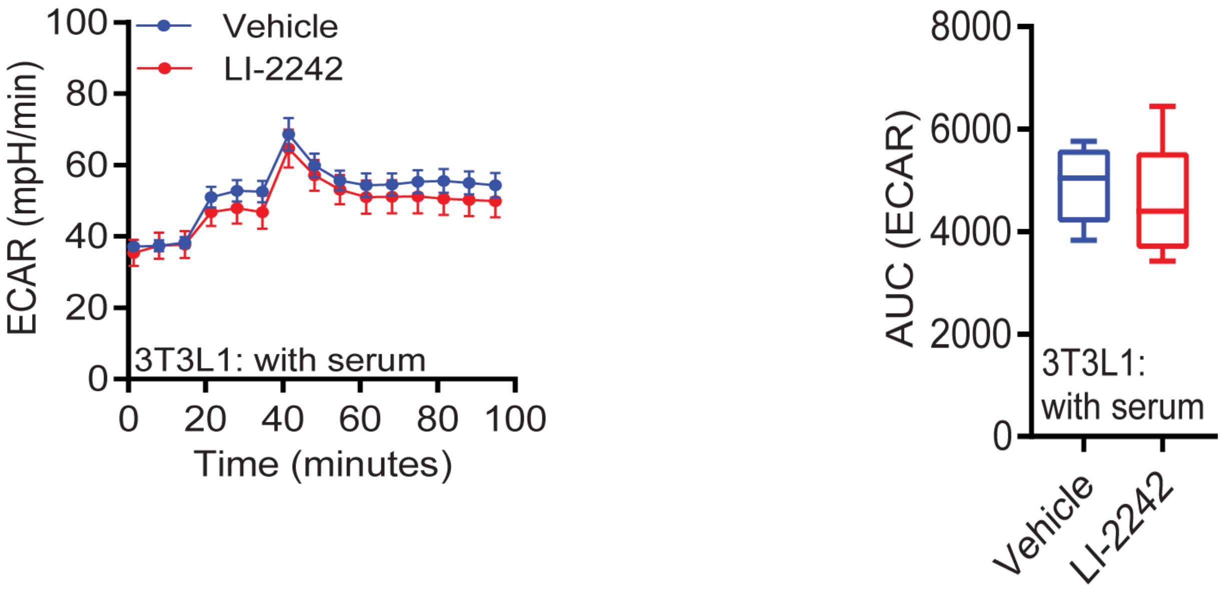

Figures S5 and S6: ECAR in vehicle- and LI-2242 [1 μM]-treated 3T3L1 adipocytes under serum containing conditions. Data represents an average of 7 individual wells.

Tables S1 and S2

**Formulation: 10 mg/kg, i.p. for 3 days (vehicle: 20% PEG 400 in PBS 0.2M, pH 7.4) N=3 mice**			
Time (h)	Mean liver conc. ng/ml (μM)	SD (ng/ml)	CV (%)
24	1605 (4)	397	24.7

**Formulation: 20 mg/kg, single i,.p. (vehicle - DMSO:Tween 80:water, 0.5:1:8.5) N=3 mice**				
Plasma PK	Unit	Mean	SD	CV (%)
$T_{1/2}$	h	5.58	0.26	4.7
T_{max}	h	0.67	0.29	43.3
C_{max}	ng/mL	70079	23235	33.2
AUC_{last}	h*ng/mL	490188	199743	40.7
AUC_{Inf}	h*ng/mL	515497	215070	41.7
AUC_%Extrap_obs	%	4.68	0.85	18.3
MRT_{Inf}_obs	h	6.97	0.39	5.56
AUC_{last}/D	h*mg/mL	24509	9987	40.7

Tables S1 and S2: Pharmacokinetic of LI-2242.

Supplementary materials

Primer sequences used for qRT-PCR:

qRT-PCR primers	Forward	Reverse
F4/80	GGATATGGAAACTTCAACTGCAA	CAAGTGTACAGAAGGAAGCATAAC
Cd11c	CAAATAGGTGGCCTCTACAAATG	GTAGGACCACAAGCCAACA
Tnfα	AGACCCTCACACTCAGATCA	GAGTAGACAAGGTACAACCCATC
Cd36	GGATGGTTTCCTAGCCTTTCA	GTGGCCCGGTTCTAATTCA
Ucp1	GTCAACACTTTGGAAAGGGAC	CAACAAGAGCTGACAGTAAATGG
Pgc1α	AGAAGCAGAAAGCAATTGAAGAG	AACGGTAGGTGATGAAACCATAG
Pparα	TGTGAAGGCTGTAAGGGCT	CTTGGCATTCTTCCAAAGCGAAT
PRDM16	CTTTGGATGGGAGATGCTGAC	CTACACGGATGTACTTGAGCC
AdipoQ	TGTTCCTCTTAATCCTGCCCA	CCAACCTGCACAAGTTCCCTT
Hprt1	CAAACTTTGCTTTCCCTGGT	TCTGGCCTGTATCCAACACTTC
Acaca	CACCTGAAGACCTTAAAGCCAA	CAGCCCACACTGCTTGTA
Fasn	CACTATACTACCCAAGACAGGAACC	GGTCGAATAACTTGGAGTTCGG
Agpat1	TCACCCAGGATGTGAGAG	GAAGTCTTGATAGGAGGACATGA
Gpat1	TGTGCTACCTTCTCTCTAACGAA	ATCTTCCTGGTCATCTTGCTCTG
Mogat1	TGGTGCCAGTTTGGTTCCAG	TGCTCTGAGGTCGGGTTCA
Plin2	GACCTTGTGTCCTCCGCTTAT	CAACCGCAATTTGTGGCTC
Plin3	ATGTCTAGCAATGGTACAGATGC	CGTGGAACTGATAAGAGGCAGG
Cidea	GCCGTGTTAAGGAATCTGCTG	TGCTCTTCTGTATCGCCCAGT
Pparγ	TGTGGGGATAAAGCATCAGGC	CCGGCAGTTAAGATCACACCTAT
Cpt1a	GAGGAACTCAAACCTATTCGTCT	GTAGAGCCAGACCTTGAAGTAA
Rplp0	AGATTCGGGATATGCTGTTGGC	TCGGGTCCTAGACCAGTGTTC

Reagents, chemicals, assay kits etc.:

REAGENT or RESOURCE	SOURCE	IDENTIFIER
**Antibody**		
p-Akt (S473)	Cell Signaling Technology	4060
Akt	Cell Signaling Technology	7631
GAPDH	Sigma Aldrich	G8795
**Reagents, chemicals, assay kits etc.**		
DMEM	Gibco	11995-065
Penstrep	Gibco	15070-063
Amphotericin B	Cayman	1397-89-3
DMEM	Gibco	A1443001
Insulin	Novo Nordisk	0169-1834-11
Glucagon	Cayman	24204
Glucose	Amresco	50997
Pyruvate	Fisher Scientific	113246
Collagenase IV	Sigma Aldrich	C5138
Collagen I, rat tail	Corning	354236
Protein ladder	Thermo Fisher	BP3603-1
Prestained Protein Standards	Biorad	1610375

Protease+phosphatase inhibitor tablets	Thermo Fisher/Pierce	A32961
Stripping buffer	Thermo Scientific	21059
Mito Stress Test Kit	SeaHorse XF	103708-100, 102601-100
RNeasy Lipid Tissue Mini Kit	Qiagen	74804
High-Capacity RT Kit	Thermo Fisher	4368814
Power SYBR Green PCR Master Mix	Thermo Fisher	4367659
AST assay kit	Teco Diagnostics	A559-150
ALT assay kit	Teco Diagnostics	A524-150
TAG assay kit	Teco Diagnostics	T532-480
Insulin assay kit	Crystal Chem	90080
Special diet		
High fat diet	TestDiets	58Y1 (based on D12492 of ResearchDiets)

**Software and Algorithms**		
GraphPad Prism 8.2.1	GraphPad Software	https://www.graphpad.com/scientific-software/prism/
ImageJ	NIH	https://imagej.nih.gov/ij/
Adobe Photoshop and Illustrator	Adobe Inc.	https://www.adobe.com/creativecloud.html
